# Comparing outcomes of single-port insufflation endoscopic breast-conserving surgery and conventional open approach for breast cancer

**DOI:** 10.1186/s12957-022-02798-6

**Published:** 2022-10-06

**Authors:** Fang Xie, Zi-Han Wang, Shan-Shan Wu, Tian-Ran Gang, Guo-Xuan Gao, Xiang Qu, Zhong-Tao Zhang

**Affiliations:** 1 General Surgery Department, Beijing Friendship Hospital, Capital Medical University, 95 Yong-an Road, Xi-Cheng District, Beijing, 100050 China; 2 Beijing Hospital of Traditional Chinese Medicine, Capital Medical University, 23 Mei shu guan Back Street, Dong-Cheng District, Beijing, 100010 China; 3 Department of Breast Disease, Peking University People’s Hospital, 11 Xi zhi men South Street, Xi-Cheng District, Beijing, 100044 China; 4 Department of Clinical Epidemiology and Evidence-Based Medicine, Beijing Friendship Hospital, 95 Yong-an Road, Xi-Cheng District, Beijing, 100050 China

**Keywords:** Breast cancer, Breast-conserving surgery, Endoscopic, Single-port, BREAST-Q scale

## Abstract

**Background:**

In the surgical treatment of breast cancer, the goal of surgeons is to continually create and improve minimally invasive surgical techniques to increase patients’ quality of life. Currently, routine breast-conserving surgery is often performed using two obvious incisions. Here, we compare the clinical efficacy and aesthetic outcomes of a novel technique using one incision, called ‘single-port insufflation endoscopic breast-conserving surgery’ (SIE-BCS), vs. conventional breast-conserving surgery (C-BCS) in patients with early-stage breast cancer.

**Methods:**

A total of 180 patients with stage I or stage II breast cancer participated in this study, of whom 63 underwent SIE-BCS and 117 underwent C-BCS. Logistic regression analysis was conducted to assess the risk of local recurrence and metastasis. Aesthetic outcomes were evaluated using the BREAST-Q scale.

**Results:**

The mean operation time was significantly longer for SIE-BCS (194.9 ± 71.5 min) than for C-BCS (140.3 ± 56.9 min), but the mean incision length was significantly shorter for SIE-BCS than for C-BCS (3.4 ± 1.2 cm vs. 8.6 ± 2.3 cm). While both surgeries yielded similar BREAST-Q ratings for satisfaction with breasts and sexual well-being, SIE-BCS was associated with significantly better ratings for physical well-being (chest area) and psychological well-being. Additionally, SIE-BCS was associated with decreased rates of adverse effects of radiation. The preliminary analysis showed that SIE-BCS did not increase the risk of local recurrence or metastasis.

**Conclusion:**

The novel single-port insufflation endoscopic assisted BCS technique is feasible, safe, and improves patients’ postoperative comfort and psychological well-being, as compared to the conventional technique.

## Background

Breast cancer is the most common malignancy among women worldwide [1–3]. Since surgery remains the mainstay of treatment for breast cancer, minimally invasive procedures are continually being created and improved upon. Indeed, advanced techniques, such as sentinel lymph node biopsy and endoscopic procedures, have led to the increased adoption of minimally invasive breast-conserving therapies (over mastectomy) by patients diagnosed with breast cancer. Importantly, breast-conserving surgery (BCS) provides faster recovery and better body image satisfaction, with survival rates similar to those obtained using mastectomy [4–7].

Conventional BCS (C-BCS) is frequently performed by creating two incisions, one to facilitate local extended lumpectomy and the other to facilitate sentinel lymph node biopsy or axillary lymph node dissection. These incisions are a drawback of the procedure, as they can cause patients to experience dissatisfaction with their visible cosmetic outcome and self-perceived body image. To overcome this unresolved and ongoing detriment to BCS, we designed an improved surgical technique called single-port insufflation endoscopic BCS (SIE-BCS), which combines the advantages of being minimally invasive and breast-conserving while avoiding the creation of obvious incision wounds and scarring on the breast. In our experience, SIE-BCS provides improved aesthetic outcomes and equal clinical efficacy as compared to C-BCS. The present retrospective cohort study involving 180 breast cancer patients treated at Beijing Friendship Hospital over a 43-month period aimed to compare the clinical outcomes of SIE-BCS and C-BCS.

## Methods

### Ethics statement

All surgical procedures and details of informed consent were approved by the ethics committee of Beijing Friendship Hospital, Capital Medical University, Beijing, China (2016-P2-070-02).

### Patient selection

We searched our hospital’s medical database for adult patients (age ≥ 18 years) who had undergone surgical treatment for stage I or II invasive breast cancer between March 2017 and July 2019. Inclusion in this study was based upon the following detailed clinical and pathological criteria: tumor constrained to the mammary gland (as confirmed by magnetic resonance imaging [MRI]); lesion diameter ≤ 3 cm; distance between the lesion and the nipple-areola complex > 3 cm; free axillary lymph nodes (i.e., not significantly fused with the axillary vein or brachial plexus nerves); adequate glandular volume; Eastern Cooperative Oncology Group grade 0–2; and normal liver, kidney, and bone marrow function. Patients were excluded if they met any of the following criteria: comorbid cardiovascular disease, myocardial infarction, or cerebrovascular disease; any condition precluding general anesthesia or surgical treatment; history of another tumor within the past 5 years; pregnancy or lactation; tumor invading the skin or subcutaneous tissue (as confirmed by MRI or physical examination); persistently positive pathological margins; and risk of failure of postoperative radiological therapy. In addition, patients with widespread disease were excluded since local excision of a single region or segment of the breast tissue would be unable to achieve negative margins with a satisfactory cosmetic result. Ultimately, the patient cohort included 63 breast cancer patients who underwent SIE-BCS and 117 patients who underwent C-BCS (Table 1). The patients were followed up until May 26, 2022.

**Table 1:** Clinical data of the 180 study patients

Variable	SIE-BCS (***N*** = 63)	C-BCS (***N*** = 117)	***p*** value
Age (years)			0.961
Mean	52.8 ± 8.7	54.0 ± 10.4	
Range	35.0–66.0	19.0–70.0	
Premenopausal, *n* (%)	24 (38.1%)	41 (35.0%)	0.770
Neoadjuvant chemotherapy, *n* (%)	7 (11.1%)	10 (8.5%)	0.381
cTNM stage, *n* (%)			0.510
I	32 (50.8%)	49 (41.9%)	
IIA	22 (34.9%)	47 (40.1%)	
IIB	9 (14.3%)	21 (18.0%)	
PR, *n* (%)			0.061
Positive	47 (74.6%)	71 (60.7%)	
Negative	16 (25.4%)	46 (39.3%)	
ER, *n* (%)			0.619
Positive	51 (80.9%)	91 (77.8%)	
Negative	12 (19.1%)	26 (22.2%)	
HER2, *n* (%)			0.011
Positive	5 (7.9%)	27 (23.1%)	
Negative	58 (92.1%)	90 (76.9%)	
Tumor location, *n* (%)			0.285
Lateral upper quadrant	42 (66.7%)	67 (57.3%)	
Lateral lower quadrant	2 (3.2%)	8 (6.8%)	
Medial upper quadrant	17 (26.9%)	31 (26.5%)	
Medial lower quadrant	2 (3.2%)	11 (9.4%)	
SLNB or ALND, *n* (%)			0.452
SLNB only	49 (77.8%)	85 (72.6%)	
SLNB and ALND	14 (22.2%)	32 (27.4%)	
pT staging, *n* (%)			0.672
pT1	45 (71.4%)	80 (73.8%)	
pT2	18 (28.6%)	37 (26.2%)	
pN staging, *n* (%)			0.517
pN0	46 (73.0%)	80 (68.4%)	
pN1	17 (27.0%)	37 (31.6%)	
Degree of tumor differentiation, *n* (%)			0.930
G1	6 (9.5%)	12 (10.3%)	
G2	52 (82.5%)	94 (80.3%)	
G3	5 (8.0%)	11 (9.4%)	
Tumor type, *n* (%)			0.731
Invasive ductal carcinoma	54 (85.7%)	102 (87.2%)	
Mucinous carcinoma	3 (4.8%)	3 (2.6%)	
Preinvasive carcinoma	6 (9.5%)	12 (10.2%)	
Tumor size (cm)			0.440
Mean	1.90 ± 0.7	1.95 ± 0.8	
Range	0.60–3.20	0.60–4.00	

HER2 status was estimated using immunohistochemistry or in situ hybridization. Tumors were considered HER2 positive if the average immunohistochemistry showed (+++). The *HER2* gene/chromosome 17 ratio was 2, and the average *HER2* gene copy number was 6. *SIE-BCS* single-port insufflation endoscopic breast-conserving surgery; *C-BCS* conventional breast-conserving surgery; *cTNM* clinical tumor, node, and metastasis; *ER* estrogen receptor; *PR* progesterone receptor; *HER2* human epidermal growth factor receptor type 2; *SLNB* sentinel lymph node biopsy; *ALND* axillary lymph node dissection; *pT staging* pathological tumor staging; *pN staging* pathological lymph node staging

### Surgical procedures

**Fig. 1:**
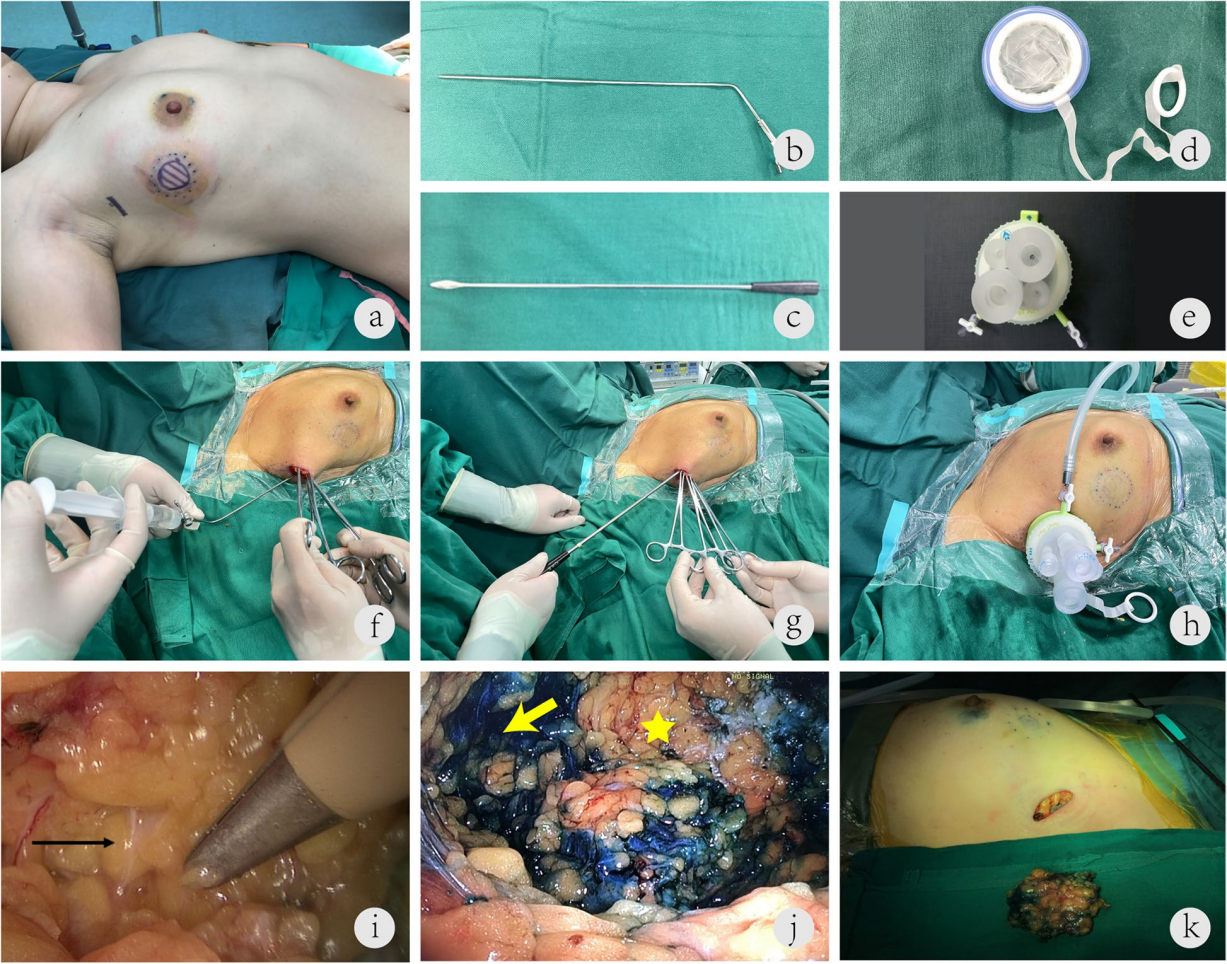
**a** Injection of methylene blue to identify sentinel lymph nodes and tumor margin. The sentinel lymph nodes were identified by injecting methylene blue into the areola. To determine the resection range, we injected methylene blue 1 cm away from the tumor margin. The single-port incision was marked along the wrinkles in the axilla. **b**, **c** Needle and tunneler used for single-port insufflation endoscopic breast-conserving surgery. **d**, **e** The single-port insufflation kit. Carbon dioxide was introduced into the body cavity to create an adequate working space. The base plate is adjustable. **f** Injection of 0.5 mg of an adrenaline solution (0.9% sodium chloride; 250 mL) into the subcutaneous layer between the skin and the mammary gland to reduce blood loss. **g** Insertion of a tunneler into the subcutaneous layer to reveal the potential space between the skin and the mammary gland. **h** Insertion of endoscopic tools from the single-port insufflation kit. **i** After insufflation, the Cooper ligament was revealed between the skin and the mammary gland. The black arrow indicates the Cooper ligament under endoscopic view. **j** Vertical sectioning from the surface of the mammary gland to the pectoral fascia. The yellow star indicates the tumor location, and the yellow arrows show the dissection range, which was marked using methylene blue. **k** The single-port incision and the specimen

All surgeries were performed by a single surgeon (Dr. Xiang Qu) with the patients in a supine position and the ipsilateral arm in abduction at 90°. In general, 0.2 mL methylene blue was first injected around the areola to identify the sentinel lymph node, According to preoperative MRI and breast ultrasound results, we palpated the tumor edge and then injected 5 mL methylene blue 1 cm away from the tumor margin to confirm the extent of tumor resection endoscopically (Fig. 1a). After 15 min, a 2.5-cm single-port incision was made along the wrinkles in the axilla for sentinel lymph node biopsy. Intraoperative frozen-section pathological examination was used to determine whether axillary lymph node dissection was necessary. If the patient required axillary lymph node dissection, we would extend the original incision to approximately 4 cm. This part of the surgery was performed under direct vision.

#### SIE-BCS procedure

We used a reusable laparoscopy equipment kit (Olympus Optical Co., Tokyo, Japan) that included optical and endoscopic instruments. SIE-BCS was performed via the same short axillary incision that was used to perform the sentinel lymph node biopsy. First, we subcutaneously inserted a water injection needle (Jinzhong Co., Shanghai, China Fig. 1b) through the axillary incision and advanced it up to the area to be resected; we then injected approximately 100 mL of a normal saline solution containing epinephrine (dilution, 1:500) between the skin and the mammary gland in order to reduce blood loss . Second, we inserted a tunneler (Jinzhong Co., Shanghai, China; Fig. 1c) into the subcutaneous layer to reveal the potential space between the skin and the mammary gland. Third, we created an adequate working space by insufflating carbon dioxide at a rate of 8 L/min (pressure, 8 mmHg) through the single-port insufflation kit (HTKD-Hang T Port, Beijing, China; Fig. 1e), which included 4 plastic trocars on its surface. After insertion of endoscopic tools from the single-port insufflation kit, we dissected the Cooper ligament with endoscopic tissue scissors until the overlying skin and the mammary gland were completely separated (Fig. 1i). Next, we used an electrocautery to vertically section the mammary gland from its anterior surface to the pectoral fascia. The excision range was determined using the prior methylene blue injection (Fig. 1j). Finally, the retromammary space was separated with the electrocautery, and the pectoral muscle fascia was removed endoscopically. To prevent metastasis from the incision, we removed the tumor and surrounding tissues by using the tools provided in the single-port insufflation kit (Fig. 1k).

Intraoperative cryosection margin evaluation was performed to confirm the extent of tumor infiltration. If the result implied residual tumor cells, then additional resection and cryosection evaluation was performed until no residual tumor cells were found. If the cryosection pathology was positive more than twice, then a mastectomy was performed immediately.

Titanium clips were placed around the incision margin of the tumor to guide subsequent radiotherapy. A running suture of absorbable monofilament 3-0 barbed thread was used to close the residual cavity of the mammary gland. The surgical field was irrigated, and one drain was placed. The single axillary incision was sutured intradermally with a 4-0 absorbable suture. All 63 patients who underwent SIE-BCS also underwent postoperative radiotherapy, according to the National Comprehensive Cancer Network guidelines [8].

#### C-BCS procedure

First, we performed a sentinel lymph node biopsy through an axillary incision. In addition, a second incision was made on the skin over the tumor surface following the Langer lines. The tumor and the surrounding glandular tissues (within 1 cm of the lesion) were removed. The excisional area encompassed the mammary gland down to the pectoral fascia. After that, an intraoperative cryosection margin evaluation was performed. If the pathological findings indicated the presence of residual tumor cells, the resection was extended until the pathological results indicated that the incised margin was negative. Then, titanium clips were placed in the residual cavity to facilitate localizing the tumor area during postoperative radiotherapy. Finally, the surgical field was irrigated, and one drain was placed; the incision was sutured layer by layer.

### Cosmetic evaluation and patient satisfaction

The aesthetic satisfaction of the patients was evaluated using the BREAST-Q scale [9, 10] at 6 months after the operation. BREAST-Q is a measurement tool that can reveal a patient’s postoperative quality of life and satisfaction with surgery. The BREAST-Q includes questions about the adverse effects of radiation, the patient’s psychosocial well-being, their physical well-being regarding the chest area, satisfaction with their breasts, and their sexual well-being. We compared the BREAST-Q scores between the two study groups.

### Statistical analysis

Measurement data were expressed as mean ± standard deviation and range. Differences in patient age, tumor size, operation time, hospitalization duration, incision length, BREAST-Q scores, blood loss, total drainage volume, and drainage duration were determined using the independent-samples *t* test (normal distribution) or the Mann-Whitney *U* nonparametric test (non-normal distribution). Count data, such as lesion site, tumor stage, and hormone receptor status, were presented as absolute numbers and percentages. The Fisher exact test was used to determine between-group differences in the intraoperative cryosection results, and rates of local recurrence and metastasis. Differences in the baseline data between the two groups were compared using the chi-square test. The association of type of surgical technique with local recurrence and metastasis was analyzed using logistic regression after adjustments for operation type, tumor size, patient age, and human epidermal growth factor receptor type 2 (HER2) status. SPSS version 22.0 software (IBM Corp., Armonk, NY, USA) was used for all statistical analyses. The threshold for a significant difference was set at *p* < 0.05.

## Results

No insufflation-related complications, such as subcutaneous emphysema, were observed. In the SIE-BCS group, 49 patients underwent sentinel lymph node biopsy only, and 14 patients underwent axillary dissection in addition to sentinel lymph node biopsy. In the C-BCS group, 85 patients underwent sentinel lymph node biopsy, and 32 patients underwent sentinel lymph node biopsy plus axillary dissection (Table 1). The mean incision length was significantly shorter in the SIE-BCS group than in the C-BCS group (3.4 ± 1.2 cm *vs*. 8.6 ± 2.3 cm, *p* < 0.001), while the operation time was significantly longer in the SIE-BCS group (194.9 ± 71.5 min) than in the C-BCS group (140.3 ± 56.9 min, *p* < 0.001). The cost of hospitalization was significantly higher in the SIE-BCS group (44,067.1 CNY) than in the C-BCS group (41,693.9 CNY, *p* < 0.05; Table 2). None of the patients in the SIE-BCS group showed positive margins during cryosection analysis, while 2 patients in the C-BCS group showed positive margins and required an additional resection to achieve negative margins (0 vs. 1.7%, *p* > 0.05; Table 2).

**Table 2:** Perioperative features of patients

Variable	SIE-BCS (***N*** = 63)	C-BCS (***N*** = 117)	***p*** value
Operation time (min)			< 0.001
Mean	194.9 ± 71.5	140.3 ± 56.9	
Range	59.0–410.0	21.0–400.0	
Intraoperative blood loss (mL)			0.701
Mean	24.3 ± 19.3	20.9 ± 15.2	
Range	5.0–00.0	2.0–50.0	
Margins during cryosection analysis, *n* (%)			0.542
Positive	0	2 (1.7%)	
Negative	63 (100%)	115 (98.3%)	
Tumor margin, *n* (%)			
Positive	0	0	
Negative	63 (100%)	117 (100%)	
Incision length (cm)			< 0.001
Median	3.0	8.0	
Q1–Q3	2.5–4.0	8.0–9.0	
Drainage duration (days)			0.596
Mean	4.3 ± 1.8	4.4 ± 1.7	
Range	1.0–10.0	2.0–8.0	
Total drainage volume (mL)			0.041
Mean	124.5 ± 102.0	87.5 ± 66.6	
Range	14.0–517.0	8.0–250.0	
Local recurrence, *n* (%)	2 (3.2%)	2 (1.7%)	0.916
Metastasis, *n* (%)	2 (3.2%)	9 (7.7%)	0.378
Hospitalization cost (yuan)			
Median	44,067.1	41,693.9	0.003
Q1–Q3	41,552.9–47,015.4	35,208.9–45,860.3	

*SIE-BCS* single-port insufflation endoscopic breast-conserving surgery, *C-BCS* conventional breast-conserving surgery

Some patients in both groups experienced local recurrence and metastasis during the 43-month follow-up (Table 2). The rates of these secondary malignancies did not significantly differ between the two surgeries. Furthermore, when the surgery type, tumor size, patient age, and HER2 status were evaluated using logistic regression modeling for their potential as risk factors for the combined events of local recurrence and metastasis, none were found to be statistically associated with these events among our patient population (Table 3).

**Table 3:** Logistic regression analysis of multiple factors potentially associated with combined events of local recurrence and metastasis

Variable	Wald	***p*** value	OR	95% CI
SIE-BCS	0.036	0.849	0.886	0.255, 3.084
Tumor size	1.270	0.260	0.969	0.917, 1.024
Age	0.450	0.503	1.272	0.629, 2.573
HER2 positivity	0.222	0.637	1.394	0.350, 5.548

*OR* odds ratio, *CI* confidence interval, *SIE-BCS* single-port insufflation endoscopic breast-conserving surgery, *HER2* human epidermal growth factor receptor type 2

All our patients were assessed for personal subjective measures of psychological, physical, and sexual well-being at 6 months after their respective surgeries, via the BREAST-Q questionnaire. Compared to the patients who had undergone C-BCS, those who had undergone the novel SIE-BCS reported significantly better satisfaction scores for physical well-being regarding the chest area and psychological well-being. In addition, the adverse effects of radiation had less impact on the patients in the SIE-BCS group than on the patients in the C-BCS group. Patients in both groups reported statistically similar satisfaction rates for the physicality of their breasts and their sexual well-being (Table 4).

**Table 4:** BREAST-Q scale scores of patients

	SIE-BCS group (***N*** = 63)	C-BCS group (***N*** = 117)	***p*** value
Adverse effects of radiation	65.1 ± 29.1	51.7 ± 22.4	0.03
Physical well-being: chest	85.2 ± 19.8	64.7 ± 15.8	< 0.001
Psychological well-being	86.9 ± 16.4	75.2 ± 19.2	0.006
Satisfaction with breasts	73.7 ± 18.3	68.8 ± 15.9	0.120
Sexual well-being	61.6 ± 28.1	64.1 ± 24.0	0.600

BREAST-Q® version 2.0 © Memorial Sloan Kettering Cancer Center and The University of British Columbia, 2017. *SIE-BCS* single-port insufflation endoscopic breast-conserving surgery, *C-BCS* conventional breast-conserving surgery

## Discussion

Although BCS is preferred for patients with early-stage breast cancer [11, 12], the conventional open technique often requires two surgical incisions, and the consequent scarring can negatively affect the postoperative aesthetics [13–15]. Indeed, patients’ postsurgical self-perception of health-related quality of life is being increasingly recognized as an important clinical outcome and considered in the therapeutic management of breast cancer. Integrating endoscopic technology in BCS, particularly for the treatment of early breast cancer, is a promising approach to respect these patients’ needs [16–19]. Herein, we describe our attempt at developing and implementing the novel SIE-BCS, which is a scarless technique, to improve disease management as well as patient satisfaction with the aesthetic outcome.

Previously, we had created an improved surgical technique to generate an adequate working space by using the skin-take-up kit [20]. For the newly created SIE-BCS technique, we found that the addition of carbon dioxide insufflation benefited the surgeon by greatly increasing the work space available to the surgical team, which facilitated their ability to perform the surgery through a single inconspicuous incision. In the report by Lai et al. [21], the method of creating the surgical space is slightly different from ours. Ozaki et al. [22] reported a similar procedure. In our experience, the work space created using carbon dioxide insufflation is better than that created using the hanging method.

In our clinical application of the novel SIE-BCS technique, all attempts were successfully completed, and there were no conversions to open surgery. Moreover, we did not find residual tumor cells after either breast-conserving technique (SIE-BCS or C-BCS), and clear resection margins were achieved in all patients. This overall outcome of our patients is better than that reported in the literature; in general, residual tumor cells at the resection margin are found in approximately 6.3% of cases [23]. We hypothesize that the low positive residual tumor cell rate in our study is attributable to the way our group determines the resection range. Before surgery, we palpate the tumor edge, add a 1-cm margin around the edge, and inject methylene blue along this margin, which is then used as the boundary for surgical resection. Our collective experience (postoperative gross pathology data not shown) indicates that the above method provides a resection size larger than the actual tumor, as compared to other detection methods, such as ultrasound examination.

**Fig. 2:**
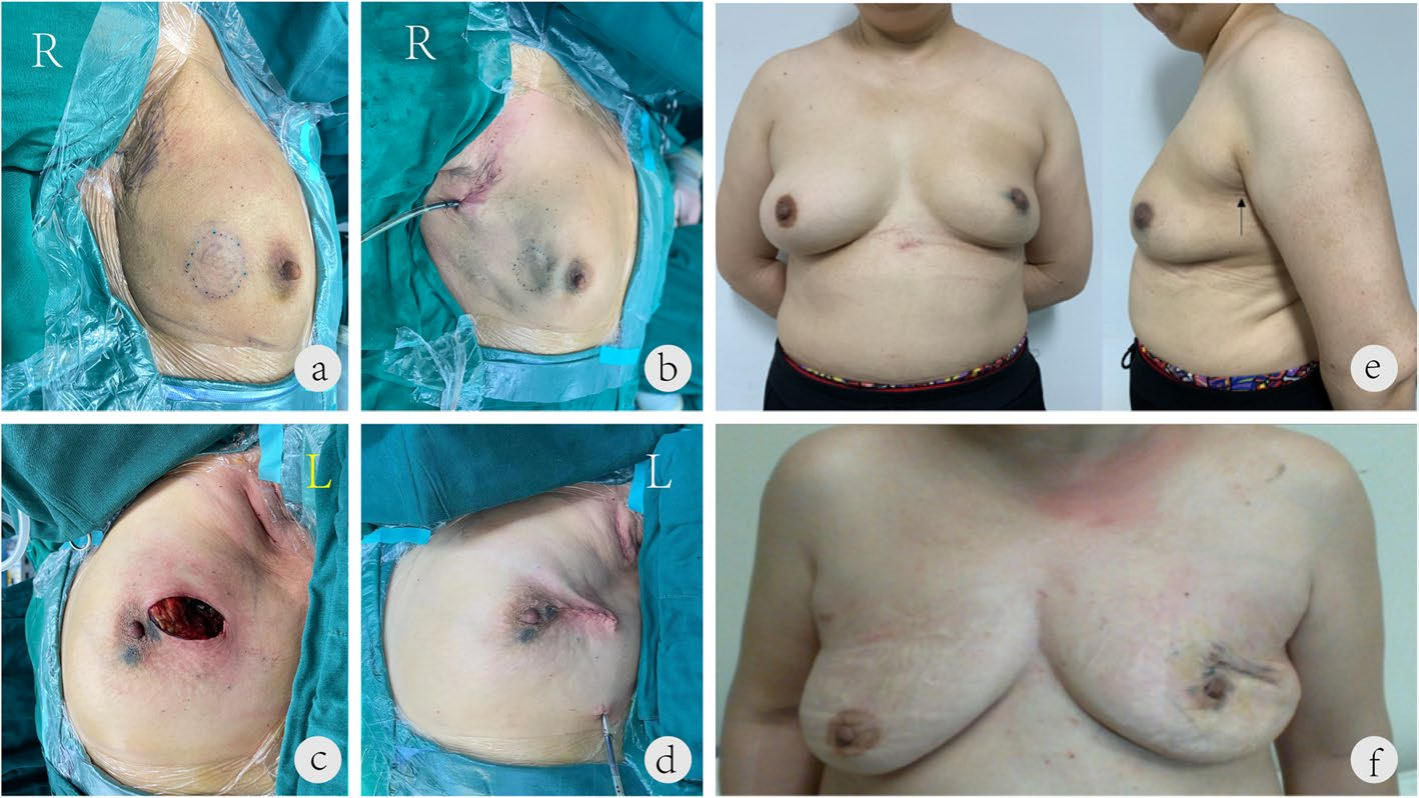
Images of incisions created during the single-port insufflation endoscopic breast-conserving surgery and conventional breast-conserving surgery. **a**, **b** The single-port incision is hidden by the upper limb and axillary fossa. The elegant appearance after the operation is evident from the front and lateral photographs. No scar is observed from the front. The black arrow indicates the hidden single-port scar in the lateral photo. **c**, **d** Routine breast-conserving surgery leaves two obvious incisions on the axillary fossa and the breast surface. **e** A 55-year-old woman with left breast cancer in the upper outer quadrant. The operation method was lumpectomy plus sentinel lymph node biopsy. Postoperative follow-up photographs taken at 12 months are shown. **f** A 68-year-old woman with left breast cancer in the upper outer quadrant. The operation method was lumpectomy plus sentinel lymph node biopsy. Postoperative follow-up photographs taken at 12 months are shown

From an aesthetic standpoint, the greatest benefit of the novel single-port endoscopic technique is related to its physical outcome. Requiring fewer incisions of shorter length than C-BCS, the SIE-BCS provides an aesthetic benefit, and this was further improved by the technique of following the natural wrinkles of the axilla to create the single-port incision, which is then hidden by the upper limb and the axillary fossa (Fig. 2a, b, and e). C-BCS typically requires two incisions on the surface of the breast (Fig. 2c, d, and f), and the readily visible scaring greatly reduces aesthetic satisfaction, with a greater potential for a negative impact on psychological well-being even after the cancer diagnosis is resolved. In other reports [21], double incisions around the axilla and areola were the most common incisions, accounting for 79% of surgeries. All our patients in the SIE-BCS group had a single axillary incision, so there was no surgical incision on the breast surface, which is a novel advantage of this technique. From a clinical standpoint, the SIE-BCS technique increased the operation time as compared to conventional surgery [24]; this phenomenon has been reported extensively, and examples of this can be found in gastrointestinal [25], hepatobiliary [26], and thyroid surgeries [27]. However, our clinical care teams do not begrudge the additional time and effort, in light of the benefits to the postoperative well-being of the patients (personal communications). Nonetheless, consideration of the increased operative cost due to the longer time and additional effort (surgeon skills, operative tools, etc.) is necessary, especially in countries where medical care costs fall on the patients or on insurance providers that operate for profit.

Another important finding in our comparison of the novel SIE-BCS technique and C-BCS is the different HER2 positivity rates between the two patient groups. Logistic regression analysis showed that choosing SIE-BCS did not increase the risk of local recurrence or metastasis. While HER2 positivity rate, tumor size, and age were not identified as independent risk factors for local recurrence and metastasis in our study cohort, other studies have identified these as risk factors for recurrence in patients undergoing endoscopic BCS [28, 29]. This inconsistency may be due to our smaller sample size and shorter follow-up period relative to those of the other studies. We have designed a prospective study to overcome these limitations. We hope that the completion of this prospective study will provide stronger and more extensive evidence for how the novel SIE-BCS technique can best be implemented for a wider range of patients to provide maximal benefit to their postsurgical health-related quality of life.

The BREAST-Q scores reported by our patients at 6 months after the surgery revealed a significantly higher satisfaction with physical well-being related to the chest area and psychological well-being after the SIE-BCS technique, which produced less scarring on the breast surface. Thus, the SIE-BCS technique may support a breast cancer survivor’s ability to attain a higher quality of life after the resolution of their diagnosis. Unexpectedly, we also observed that the SIE-BCS group had a lower rate of adverse effects of radiation than the C-BCS group. By removing one incision, the SIE-BCS can avoid scarring on the breast surface that may otherwise have caused discomfort from scar contracture during radiotherapy. The reduction of the side effects of radiotherapy and improvement in physical well-being related to the chest area likely contributed to the improvement in psychological well-being experienced by the patients who underwent SIE-BCS. As an aside, the full cohort of patients in our study expressed breast satisfaction and sexual well-being, with no significant differences in these two parameters between the two study groups. Although SIE-BCS has the advantage of avoiding incisions on the breast surface, this advantage may not be reflected in the Breast-Q scores because Breast-Q was not designed for the comparison for newly developed minimal-access breast cancer operations, such as SIE-BCS. Most of the questions in the “Satisfaction with breast” section were to do with the appearance of the breasts in clothes or bras, rather than the scar length, appearance, and location. Thus, the high “Satisfaction with breast” scores in both study groups may be related to the patients’ general satisfaction with having successfully undergone BCS.

## Conclusion

In this study, we introduced a novel single-port endoscopic technique for BCS called SIE-BCS that reduced the length and number of incisions on the affected breast. Logistic regression analysis showed that SIE-BCS did not increase the risk of local recurrence and metastasis. Moreover, SIE-BCS-treated patients reported significantly better scores for adverse effects of radiation, physical well-being for chest self-perception, and psychological well-being compared to C-BCS-treated patients. Because this study was retrospective with a short follow-up time, we have designed a prospective study to further demonstrate the feasibility and success of this novel breast-conserving technique. This novel technique of using insufflation and a single-port could be a safe and advantageous option for breast cancer patients who are candidates for BCS.

## Data Availability

The datasets generated and analyzed during the present study are available from the corresponding author on reasonable request.

## Abbreviations

C-BCS: Conventional breast-conserving surgery
CI: Confidence interval
OR: Odds ratio
SIE-BCS: Single-port insufflation endoscopic breast-conserving surgery
